# Exosome-mediated metabolic reprogramming: the emerging role in tumor microenvironment remodeling and its influence on cancer progression

**DOI:** 10.1038/s41392-020-00359-5

**Published:** 2020-10-19

**Authors:** Enli Yang, Xuan Wang, Zhiyuan Gong, Miao Yu, Haiwei Wu, Dongsheng Zhang

**Affiliations:** 1 Department of Oral and Maxillofacial Surgery, Shandong Provincial Hospital Affiliated to Shandong First Medical University, 324 Jingwu Road, 250021 Jinan, China; 2 Department of Oral and Maxillofacial Surgery, Shandong Provincial Hospital Affiliated to Shandong University, 324 Jingwu Road, 250021 Jinan, China

**Keywords:** Cancer microenvironment, Cancer metabolism

## Abstract

Metabolic reprogramming is reported to be one of the hallmarks of cancer, which is an adaptive mechanism by which fast-growing cancer cells adapt to their increasing energy demands. Recently, extracellular vesicles (EVs) known as exosomes have been recognized as crucial signaling mediators in regulating the tumor microenvironment (TME). Meanwhile, the TME is a highly heterogeneous ecosystem incorporating cancer cells, fibroblasts, adipocytes, endothelial cells, mesenchymal stem cells, and extracellular matrix. Accumulated evidence indicates that exosomes may transfer biologically functional molecules to the recipient cells, which facilitate cancer progression, angiogenesis, metastasis, drug resistance, and immunosuppression by reprogramming the metabolism of cancer cells and their surrounding stromal cells. In this review, we present the role of exosomes in the TME and the underlying mechanism of how exosomes exacerbate tumor development through metabolic reprogramming. In addition, we will also discuss the potential role of exosomes targeting metabolic process as biomarkers for tumor diagnosis and prognosis, and exosomes-mediated metabolic reprogramming as potential targets for cancer therapy. Furthermore, a better understanding of the link between exosomes and metabolic reprogramming, and their impact on cancer progression, would provide novel insights for cancer prevention and treatment in the future.

## Introduction

In the past decade, the cancer metabolism has received enormous interest and wide attention. A common metabolic feature of cancer cells is obtaining necessary nutrient from a nutrient-deficient environment and using the nutrient to maintain cell viability, proliferation, and build biomass. The metabolic activity of cancer cells is altered compared with normal cells, and these changes support the acquisition and preservation of malignant features. Therefore, metabolic reprogramming is regarded as one of the hallmarks of cancer.^1,2^ The abnormal metabolic functions of cancer cells were first proposed by Otto Warburg, who discovered that cancer cells consumed more glucose relative to normal cells. Besides, the rate of glycolysis in cancer cells is much higher than oxidative phosphorylation (OXPHOS), even under sufficient oxygen conditions; this phenomenon is known as the Warburg effect.^3^ Since then, a number of researches have focused on the cancer-related metabolic reprogramming in the metabolic pathway, including metabolic changes in glucose, amino acids, and lipids, to explore potential therapeutic targets in cancer progression.

Exosomes are membrane-coated vesicles, which are a subset of EVs with a 40–100-nm diameter. Exosomes are secreted by many kinds of cells into the extracellular microenvironment through the multivesicular bodies (MVBs), including cancer cells.^4^ Exosomes contain protein, mRNA, miRNA, transcription factors, lipids, and other biologically active constituents. The role of exosomes in intercellular communication is accomplished by mediating the exchange of substances between cells, thereby changing the biological properties of the recipient cells. Therefore, exosomes have emerged as essential players in extracellular communication.^5,6^ Accumulated evidence indicates that cancer cells actively release exosomes into the surrounding microenvironment, and these vesicles have the pleiotropic capacity in regulating tumor growth and progression, neovascularization, immune escape, promoting tumor invasion, and metastasis.^7,8^ Therefore, exosomes could regulate intercellular communication not only between cancer cells, but also with the TME.^9^ Up to now, most researches focus on exosomes containing certain proteins and RNAs. Nevertheless, recent researches have shown that the components of exosomal cargo are far more complicated and contain metabolites. These molecular metabolites could be transferred to surrounding cancer cells via exosomal pathway and influence the metabolism of recipient cells to favor cancer progression.^10,11^

There is a growing body of studies that describe a relationship between exosomes and the tumor development and summarize the regulatory effects of exosomes on cancer therapy resistance, metastasis, and immunity.^12–14^ On basis of these studies, we particularly focus on the emerging role of exosome-mediated metabolic reprogramming in the regulation of the TME and cancer progression in the present review, which can provide potential anti-cancer therapeutic targets in the future research.

## Exosome and metabolic reprogramming

### Biogenesis and characteristics of exosome

**Fig. 1:**
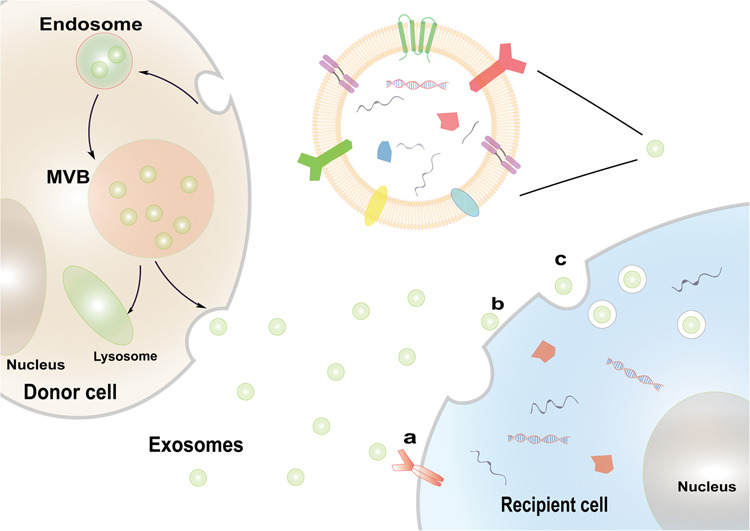
The biogenesis and characteristics of exosomes. Exosomes are secreted by donor cells into the intercellular microenvironment through the multivesicular bodies (MVBs). Exosomes can transfer biologically functional molecules to recipient cells through three ways, including **a** intercellular signaling through receptor-ligand binding, **b** exosomes directly fuse with the recipient cells membrane and release their contents, and **c** recipient cells phagocytose exosomes

The formation of exosomes is a tightly regulated process and undergoes four main processes (Fig. 1): initiation, endocytosis, MVBs formation, and secretion of exosomes.^15^

The cell membrane sags inward to form the early endosomes, with accumulating intraluminal vesicles (ILV) in their lumen. Then, early endosomes maturation lead to MVBs formation. Generally, the MVBs are degraded by fusing with the lysosome, but some MVBs are combined with plasma membrane and secreted exosomes to extracellular space.^16^ To date, the most exhaustive mechanism that described the generation of ILVs and MVBs are the ESCRT-independent or -dependent pathways.^17^ Rab guanine triphosphatase, also known as GTPase, has been considered as a vital regulatory mediator in the secretion of exosomes. Besides, the results from Ostrowski et al.^18^ demonstrated that Rab27a and Rab27b promoted the occurrence and stability of MVBs docking. Another study showed that KIBRA could regulate exosome secretion by inhibiting proteasome degradation of Rab27a, and KIBRA depletion could increase the number and size of MVBs.^19^ Furthermore, HSP90 and mTORC1 were also reported to have a regulatory effect on exosomal secretion.^20,21^

Exosomes are nano-sized extracellular vesicles, also referred to as “cargo”, which consist of a bilipid layers. Exosomes encapsulate a large number of molecules, including proteins, nucleic acids, lipids, and metabolites. This exosomal cargo is highly variable, depending on the source of cell types, the state of the cell, and its microenvironment.^17,19^ Exosomes can transfer the information to recipient cells in three different pathways: (1) The protein on the exosomal membrane directly contacts the protein on the receptor cell membrane, and then triggers the intracellular signaling cascade; (2) the exosomal membrane fuses with the recipient cells membrane and releases its contents into the recipient cell; (3) targeting cells directly phagocytose exosomes and internalize them into their components.^22^

Different approaches have been developed for the isolation and purification of exosomes based on their biological, physical, and chemical properties, including ultracentrifugation, ultrafiltration, size exclusion chromatography, and microplate-based magneto-immunocapture.^23,24^ At present, the most widely accepted technique for exosome isolation is the differential ultracentrifugation. Although no uniform identification criteria have been established for exosome isolation, differential ultracentrifugation has been proved to be effective in different studies.^25^ The detection of exosomes can be achieved by nanoparticle tracking analysis (NTA) and transmission electron microscopy (TEM); additionally, western blotting can be applied to analyze exosomal markers. The exosomal markers include ESCRT-related proteins (Tsg101 and Alix), tetraspanins (CD81, CD63, and CD9), cytoplasmic proteins (Hsp90 and Hsp70), Integrins, and Annexins.

### The metabolic reprogramming in cancer cell

During cancer progression, cancer cells should exhibit high metabolic plasticity to adapt themselves to the dynamic changes in tumor.^26^ High oxygen and nutrient utilization rate is essential to maintain heightened biosynthetic and bioenergetic demands for tumor cell proliferation and dissemination.^26^ Normally, cells mainly utilize glucose to generate ATP. Metabolism of glucose is mainly accomplished by glycolysis or tricarboxylic acid (TCA) cycle-mediated OXPHOS. Glycolysis produces less ATP per mole glucose compared to OXPHOS. In other words, glycolysis produces 2 moles ATP per mole of glucose, while OXPHOS produces 36 moles ATP per mole of glucose.^27^ Normal cells prefer to use glucose to generate pyruvate, and then utilize TCA and OXPHOS to produce ATP. On the contrary, cancer cells prefer to use glycolysis rather than OXPHOS under normoxia conditions to create energy. The increased glucose uptake and the enhanced glycolysis, as well as high production of lactate even under the aerobic condition, are considered as a hallmark of the tumor. These changes in metabolism are called “Warburg effect”. With the favor of tumor Warburg effect, energy can be rapidly produced and conducive to other metabolic pathways to produce lipids, amino acids and nucleotides for cancer cell growth.^28^

In addition to glucose, lipids, and amino acids are also important metabolites for tumor cell growth and progression. Lipid metabolism is also changed in highly proliferating cancer cells that have an increased demand for cholesterol and lipids. Carbon should be transferred from energy generation to fatty acids (FAs) for signaling lipids, membrane biosynthesis, or energy sources. In order to meet their needs, cancer cells will uptake exogenous lipoproteins and lipids to enhance de novo synthesis of FAs and cholesterol biosynthesis.^29^ FAs synthesis occurs mainly in the cytoplasm. Firstly, acetyl-CoA is converted to malonyl-CoA by acetyl-CoA carboxylase (ACC). Then, fatty acid synthase (FASN) assembles malonyl-coenzyme A to palmitate. At present, the expression of three key enzymes related to FAs synthesis, including FASN, ACC, and ATP-citrate lyase (ACLY), have been upregulated in various types of tumors. Inhibition of these enzymes can suppress tumor growth both in vivo and in vitro.^30,31^ Moreover, FA oxidation (FAO) has emerged as critical players in the cancer cells metabolism, and the upregulated FAO facilitates the proliferation and survival of cancer cells.^32^ Meanwhile, NADPH produced by FAO can make cancer cells counteract oxidative stress.^33^ Another frequent metabolic change in cancer cells is increased amino acid metabolism, especially glutamine and serine metabolism. Glutamine is a major energy substrate, and the metabolism of glutamine could produce α-ketoglutarate and TCA cycle intermediates to provide an additional energy source for cancer cells.^34^ Glutamine metabolism is influenced by multiple oncogenic signaling pathways. For example, overexpressing MYC by tumors shows a dependence on glutamine metabolism, and MYC can promote the expression of glutamine transporters and glutaminase (GLS).^35^ Serine biosynthesis has also been investigated in cancer cells. Phosphoglycerate dehydrogenase (PHGDH) is a crucial enzyme for serine synthesis, and the expression levels of PHGDH are significantly increased in breast cancer cells.^36^

With the in-depth study of tumor metabolism, the realization of metabolic reprogramming in the cancer has gone far beyond originally described “Warburg effect”. A better understanding of tumor metabolic alterations and its underlying molecular mechanisms will help us explore more effective cancer treatment. Moreover, selectively targeting the metabolic reprogramming of tumor cells will be an attractive direction for cancer treatment.

## Exosome-mediated metabolic reprogramming in the TME

**Fig. 2:**
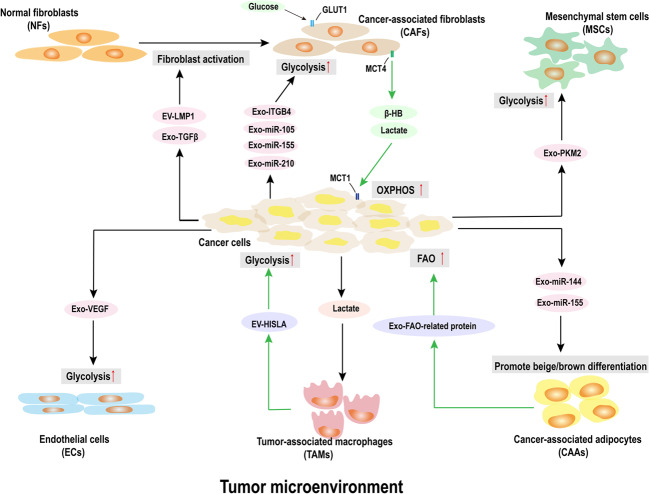
The exosome-mediated metabolic reprogramming in the tumor microenvironment. Exosome-mediated metabolic reprogramming occurs in cancer cells and their surrounding stromal cells in the TME. Stromal cells metabolic reprogramming is affected by exosomes derived from cancer cells, and acts as a feedback loop to drive metabolic changes in cancer cells or to provide metabolic resources required for cancer progression

Exosomes have been considered as critical mediators in cancer progression, where they regulate extracellular communication not only with the cancer cells but also with the stromal cells.^22^ The TME is a highly complex and heterogeneous ecosystem incorporating cancer cells, fibroblasts, adipocytes, endothelial cells, mesenchymal stem cells, and extracellular matrix (Fig. 2). It has been widely accepted that the development of TME is crucial for cancer progression.^37^ Exosome-mediated metabolic reprogramming is not limited to cancer cells, but is also found in stromal cells in the TME, which suggested the involvement of exosome-mediated metabolic reprogramming in the TME. The metabolic remodeling in stromal cells is influenced by the cancer cells and acts as a feedback loop to facilitate the growth of cancer cells.^38^ Stromal cells could drive the metabolic changes in the cancer cells and provide the metabolic resources needed for cancer progression.^38^

### Cancer-associated fibroblast

Cancer-associated fibroblasts (CAFs), as the major component in the TME, are mostly defined by morphological features or expression of markers, including α-smooth muscle actin (αSMA), fibroblast-specific protein-1 (FSP1/S100A4), and fibroblast activation protein (FAP).^39^ Normal fibroblasts could be activated during wound healing and inflammation, and these activated fibroblasts are usually termed as myofibroblasts.^40^ Similarly, the cancer development might be considered as a “cancer wound”, which could activate the fibroblasts into CAFs.^41^ CAFs are derived from different origins, mostly from normal fibroblasts, epithelial cells, mesenchymal cells, endothelial cells, epithelial-mesenchymal transition (EMT) and endothelial to mesenchymal transition (EndMT).^42^ Furthermore, CAFs are considered to promote tumor growth and progression through various pathways, such as secretion of inflammatory factors and growth factors.^42^

Recent studies showed that CAFs play a vital role in tumor growth through regulating metabolism. Cancer cells can promote glycolysis of CAFs. In turn, CAFs can provide metabolites for cancer cells and facilitate cancer cells proliferation through the TCA cycle and OXPHOS. This phenomenon was recognized as the “Reverse Warburg Effect”.^43^ Recently, the export of lactate in CAFs and the uptake of lactate in cancer cells has been confirmed in the metabolism of cancer.^44^ The lactate symporters MCT1 and MCT4 are critical regulators in establishing a lactate shuttle system. MCT4 favors export of lactate, whereas MCT1 facilitates cellular lactate uptake. Thus, the tumor that utilizes the “Reverse Warburg Effect” is featured by higher MCT4 expression in CAFs and higher MCT1 expression in tumor cells.^44^ Additionally, more recent studies indicated that Caveolin-1 (Cav-1) might contribute to cancer progression by regulating the metabolism of CAFs.^45,46^ Cav-1 is a membrane-bound scaffolding protein related to endocytosis, signaling transduction, cell migration, and the distribution of cholesterol.^45^ The reactive oxygen species (ROS) that is secreted by the cancer cells induces oxidative stress in CAFs, which leads to autophagy and causes the degradation of Cav-1 and mitochondria.^47,48^ Moreover, Michael and colleagues^49^ found that knockdown of Cav-1 resulted in significant upregulation of glycolytic enzymes, including lactate dehydrogenase and pyruvate kinase. These results further demonstrate that the metabolic symbiosis exists between cancer cells and CAFs.

In recent years, there is growing evidence that exosomes have been recognized as crucial mediators in regulating the extracellular communication and metabolic reprogramming between CAFs and cancer cells. Several studies have shown that cancer cells were capable of transforming normal fibroblasts to CAFs through exosomes.^50,51^ Webber et al.^50^ indicated that exosomes secreted by prostate cancer cells contained TGF-β and could mediate the activation of fibroblasts. Besides, another study noted that different pathological stage of colorectal cancer-derived exosomes could lead to different stages of CAF-like changes in normal fibroblasts and increased expression of CAF marker α-SMA.^51^ Meanwhile, exosome-activated fibroblasts showed significant downregulation of Cav-1 and upregulation of the glucose transporter GLUT1. Increased expression of GLUT1 enhanced the uptake of glucose and promoted the glycolysis in the activated fibroblasts.^51^ Furthermore, the vital role of cancer cells-released exosomes in remodeling fibroblasts metabolism and promoting glycolysis were also confirmed in breast cancer cells.^52,53^ Additionally, exosomes derived from cancer cells could induce the expression of MCT4 in CAFs to export β-HB and lactate into the cancer cells, and cancer cells expressing MCT1 utilize lactate to improve the level of OXPHOS.^53,54^ All these results demonstrated that exosome-mediated metabolic reprogramming plays a crucial role in the intercellular communication between cancer cells and CAFs.

### Cancer-associated adipocyte

Considering the link between cancer progression and obesity, adipocytes are identified as an essential component of the TME.^55^ Dirat et al.^56^ reported that adipocyte co-cultivated with cancer cells exhibited a changed phenotype with reduced lipids content and decreased adipocytes markers. Overexpression of IL6, IL1, and MMP11 were also detected in the activated adipocytes, which are referred to as cancer-associated adipocytes (CAAs).^56^ Then, CAAs proceed to cultivate with cancer cells and adopt a phenotypical change to generate fibroblast-like cells named adipocyte-derived fibroblasts (ADF), which may be part of the CAFs population in the desmoplastic reaction of cancer.^57^ CAAs play functional roles in the tumor aggressiveness and progression through endocrine and paracrine influences on cancer cells by secreting adipocyte-derived factors, such as leptin, adiponectin, resistin, visfatin, TNF-α, IL-6, and MCP-1.^58,59^ Conversely, Cancer cells-derived signaling molecules triggered the lipolysis in CAAs and led to adipose atrophy in the human body, which is a manifestation of the cancer cachexia.^58,60^

Lately, adipocytes were mainly considered to be great energy storage and provided high-energy metabolites in the TME.^61^ For example, CAAs released exogenous FFAs, which were taken up by cancer cells via cell surface CD36.^62^ Additionally, FFAs could provide sufficient energy for cancer cells through FAO.^63^ Thus, CAAs can regulate the metabolic reprogramming of cancer cells, which in turn promote cancer progression.

Numerous studies have shown that exosomes mediate important communication between CAAs and cancer cells. Some previous studies have indicated that breast cancer cells-derived exosomes could remodel the resident adipocytes tending to activated phenotype by promoting beige/brown differentiation and increasing the catabolism in adipocytes.^64,65^ Meanwhile, MSC-differentiated adipocytes-associated exosomes could be incorporated by breast cancer cells, which promoted the proliferation and migration of cancer cells.^66^ A study by Lazar et al.^67^ revealed that exosomes secreted from adipocytes were internalized by melanoma cells, which consequently promoted the tumor invasion and migration. Meanwhile, the proteome of exosomes showed an enrichment in proteins associated with lipid metabolism (e.g., FAO-catalyzing enzymes), which significantly enhanced FAO in melanoma cells.^67^ Similarly, another study also found that enriched FAO-related proteins were harbored in EVs from adipocytes, and melanoma cells could uptake these exosomes to fuel FAO. Intriguingly, adipocyte EVs-induced FAO is enhanced by obesity, but the process does not dependent on increased protein transfer.^68^ Consequently, it could be speculated that adipocytes-originated exosomes might exacerbate tumor development by remodeling cancer cells metabolism.

### Endothelial cell

The cancer-associated endothelial cells (CAECs) are important components in the TME. It is widely accepted that CAECs-mediated tumor angiogenesis is of vital importance for tumor progression. The neovascularization supplies nutrient and oxygen to cancer cells, which support tumor progression and provide pathways for tumor metastasis.^69^ The sustained hypoxia, combined with the secretion of cytokines, such as VEGF, facilitates tumor revascularization by triggering the remodeling of endothelial progenitor cells that are derived from bone marrow toward cancer.^70^

Glycolysis is a hallmark of tumor ECs, and the ECs in the tumor vasculature depend more on glycolysis to produce ATP than normal ECs.^71^ A hyperglycolytic phenotype was confirmed in tumor ECs, which is evidenced by the enhanced expression of the glycolytic key enzyme PFKFB3 and the glucose transporter GLUT1.^71^

Recently, research shows that exosomes secreted by cancer cells can remodel the metabolism of ECs. Wang et al.^72^ found that exosomes derived from acute myeloid leukemia (AML) cells, containing VEGF/VEGFR, could promote the glycolysis in ECs. Although recent studies have discovered the potential role of exosomes secreted by cancer cells on regulating the metabolism of ECs, few studies have focused on the feedback effects of ECs-derived exosomes on cancer cells in the tumor field. However, a research has shown that lower levels of ECs-derived microvesicles are related to better survival after chemotherapy in breast cancer patients, suggesting that ECs-derived exosomes also might have an important influence on tumor progression.^73^

### Mesenchymal stem cell

Mesenchymal stem cells (MSCs) are pluripotent cells, which can differentiate into multiple mesenchymal lineages, including mesenchymal stromal cells and multipotent stem stromal cells. The multi-lineage differentiation of MSCs determines its profound effects on the function and formation of TME.^74^ MSCs presented in tissue throughout the body, such as bone marrow, ovary, and omentum, which is advantageous for wound healing and tissue repair.^75^ In the TME, tumor-secreted factors induce tumor-promoting phenotypes in MSCs population, developing cancer-associated mesenchymal stem cells (CA-MSCs).^76^ CA-MSCs can differentiate into crucial stromal components, including CAFs and CAAs. Compared with normal tissues MSCs, CA-MSCs exhibited a high expression of TGF-β superfamily member, which is essential for cancer progression, tumor proliferation, and induction of chemoresistance.^75,76^

Little is known about the role of metabolic changes on the intercellular communication between MSCs and cancer cells in tumor progression. However, MSCs have the ability to differentiate into CAFs which influence the growth and invasion of cancer cells by secreting diverse metabolites, cytokines, and growth factors.^77^ In fact, the metabolic crosstalk between cancer cells and CAFs deeply affects TME remodeling, which critically drives tumor growth.^78^

Cancer cells can affect the metabolic reprogramming of CA-MSCs through secreted factor-mediated intercellular communication. Furthermore, exosomes play an important role in this regard. Ma et al.^79^ showed that glioma cells-derived exosomes remodeled glucose metabolism and promoted the glycolysis in MSCs, leading to their transformation of the tumor-like phenotype. Moreover, Dai et al.^80^ showed that exosomes secreted by prostate cancer containing PKM2 were transferred to MSCs, which could promote the progression of tumor metastasis. Additionally, MSCs influenced by cancer-exosomes could secret more exosomes as a feedback, which could promote tumor angiogenesis by up-regulating the level of glycolysis.^81^

### Tumor-associated macrophage

Macrophages are vital drivers of pro-tumor inflammation. Macrophages could be polarized to M1 type after stimulating by IL-12, IFN-γ, LPS, TNF-α, Toll-like receptor (TLR) agents, and have a crucial role in mediating helper T cell 1 (Th1) type immune response and anti-pathogen infection. IL-4, IL-5, IL-10, IL-13, CSF1, TGF-β1, and prostaglandin E2 (PGE2) induce macrophages to M2 type polarization that has been concerned as an important mediator in the process of tissue repairing and wound healing.^82,83^

Tumor-associated macrophages (TAMs) are one of the major immune cells in the TME. TAMs counteract the cytotoxic effects of NK cells and T cells, thereby facilitating the escape of cancer cells from immune surveillance, which plays a crucial role in cancer proliferation and migration.^38^ TAMs are highly plastic, with M1 type TAMs exhibiting immunostimulatory properties and M2 type TAMs exhibiting immunosuppressive properties.^84^ TAMs in tumor usually exhibit an M2 phenotype, which is usually related to poor prognosis.^85^ Abundant studies have demonstrated that exosomes are related to the polarization of TAMs. Exosomal miR-1246 secreted from mutp53 colon cancer cells triggered M2 type polarization of macrophages. The reprogramming TAMs promoted anti-inflammatory immunosuppression by increasing the expression of TGF-β.^86^ Similarly, pancreatic cancer cells-derived exosomes induced M2 polarization of macrophages by activating PTEN/PI3Kγ pathway.^87^ Oppositely, a few studies have also shown that tumor-derived exosomes triggered M1 polarization in macrophages.^88,89^

Importantly, macrophage polarization needs to be achieved by altering intracellular metabolism. M1 type expresses a high rate of glycolysis and requires lower oxygen supplement, which would help themselves to survive in the hypoxic microenvironment at sites of cancer and chronic inflammation. On the other hand, M2 type prefer to utilize OXPHOS and FAO.^90,91^ Furthermore, TAMs showed complex pattern changes of metabolism after influencing by different factors in the TME, including increased glycolysis, altered nitrogen cycle metabolism, and FAs synthesis.^92^

Moreover, a recent study discovered a feed-forward loop between TAMs and tumor cells through exosome-mediated metabolic reprogramming.^93^ TAMs enhance the aerobic glycolysis and apoptotic resistance of breast cancer cells via the EVs transmission of HISLA, which could block the hydroxylation and degradation of HIF-1α in tumor cells. Reciprocally, lactate released from glycolytic tumor cells upregulates HISLA in macrophages.^93^ These results suggested that exosome-mediated metabolic reprogramming is an important part of intercellular communications between immune and tumor cells.

## Exosome-mediated metabolic reprogramming in cancer progression

**Fig. 3:**
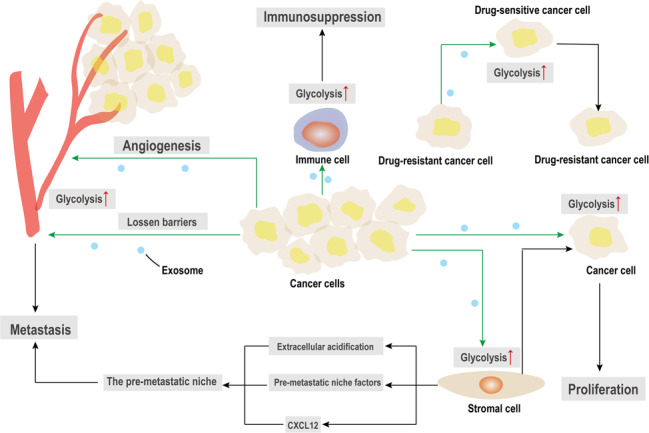
The exosome-mediated metabolic reprogramming in the cancer progression. Exosomes are vitally involved in tumor proliferation, angiogenesis, metastasis, drug resistance, and immunosuppression by metabolic reprogramming. Cancer-derived exosomes can facilitate the proliferation of surrounding cancer cells via glycolysis. Meanwhile, exosomes can be transferred into the target endothelial cells (ECs), which induced glycolysis and promoted angiogenesis activity in the ECs. Cancer cells-derived exosomes increase glycolysis in stromal cells, which contribute to create the pre-metastatic niche and facilitate the tumor metastasis. Additionally, cancer-associated exosomes can lossen the ECs barriers by remodeling metabolism, which induces vascular permeability and releases cancer cells into the bloodstream for metastasis. Moreover, exosomes secreted by drug-resistant cancer cells could be incorporated by drug-sensitive cells, thereby enhancing the glycolysis and transferring the phenotype of drug-resistant to drug-sensitive cancer cells. Notably, cancer-derived exosomes can induce immunosuppression by remodeling immune cell metabolism

A growing number of studies have shown that exosomes-mediated metabolic reprogramming has a critical role in cancer progression (Fig. 3). Exosomes deliver the biologically active substance to regulate the metabolic state of recipient cells and facilitate tumor proliferation, angiogenesis, metastasis, drug resistance, and immunosuppression. In this section, we will introduce how exosomes affect cancer progression through the regulation of metabolism.

### Tumor proliferation

Cancer cell proliferation requires a massive accumulation of intracellular substances, such as proteins and lipids, to promote cancer progression. Accumulating evidence showed that the exosomes are rich in great content to contribute to cancer cells proliferation significantly. For example, exosomal EGFRvIII mRNA derived from glioblastoma patients’ serum, which promoted the glioma cells proliferation.^94^ Another study suggested that the moderately malignant pancreatic cell (PC) line could incorporate exosomes from the highly malignant PC line, and enhanced the proliferation of the former. Furthermore, the exosomal zinc transporter (ZIP4) upregulation may be associated with the enhanced proliferation ability of recipient cells.^95^

Proliferating cancer cells often absorb nutrients that exceed bioenergy requirements and shunt metabolites to pathways that support biosynthesis. The metabolism of these cells is different from the metabolism of quiescent cells in its high rates of the aerobic glycolysis and biosynthesis of macromolecules.^96^

In a recent study, Shao and colleagues^97^ demonstrated that irradiated lung cancer cells released exosomes to other lung cancer cells, and facilitated the proliferation and growth of recipient cells. Further research showed that exosomes containing the metabolic enzymes ALDOA and ALDH3A1 promoted unirradiated lung cancer cells proliferation by accelerating glycolysis. The exosomal lncRNA SNHG3, as a sponge of miR-330-5p, positively regulated the expression of PKM, inhibited the OXPHOS, increased glycolysis, and promoted breast cancer cells proliferation.^98^ Another study found that miR-105-containing exosomes derived from breast cancer cells could induce metabolic reprogramming in the CAFs. Additionally, CAFs displayed different metabolic profiles to promote cancer cells growth according to the metabolic environment.^53^ Together, these findings indicate that exosomes are important mediators of metabolic reprogramming, thereby facilitating proliferation of cancer cells.

### Tumor angiogenesis

Angiogenesis is a complicated process by which the new microvessels are formed from existing vessels, and it is involved in both pathological and physiological processes of the body.^99^ Besides, tumor angiogenesis supply tumor with oxygen and nutrients for continuous tumor growth, metastasis, and progression.^100^

Recent studies have shown that cancer-associated exosomes exacerbate angiogenesis in cancer progression. The cargo of exosome is enriched in angiogenesis-related factors, such as angiogenic proteins, miRNAs, mRNAs, and induces the phenotype changes and functions in ECs, which results in increased ability of EC proliferation, migration, and sprouting.^101^

Glycolysis in ECs have a critical role in the process of angiogenesis, because ECs rely on glycolysis rather than OXPHOS to produce ATP for proliferation and migration.^102,103^ Moreover, PFKFB3 is a key enzyme for glycolysis in ECs, which could promote EC proliferation and angiogenic sprouting.^102^

It is well known that VEGF is one of the most important angiogenesis-related factors. Some studies have indicated that VEGF facilitates tumor angiogenesis by inducing a metabolic shift from OXPHOS to glycolysis in ECs.^104,105^ Recently, Huang et al. found that exosomes derived from AML cells could regulate vascular reprogramming in AML.^72^ VEGF-containing exosomes were transferred into the targeted ECs, and induced the upregulation of glycolytic capacity and angiogenesis activity in ECs through activating VEGF signaling.^72^ Virus-encoded microRNAs packed in exosomes from Kaposi’s sarcoma-associated herpesvirus (KSHV) infected cell were incorporated by the neighboring cells, resulting in metabolism shifted toward the glycolysis and reduced mitochondrial biogenesis in surrounding non-infected cells. The exosomes supported the growth of infected cells by mediating the metabolic remodeling of neighboring cells, thus promoting angiogenesis of Kaposi’s sarcoma.^106^ Obviously, these studies show that exosomes play a critical role in angiogenesis through transferring angiogenic factors or miRNA to regulate the angiogenic function of ECs by metabolic reprogramming.

### Tumor metastasis

Metastasis is a critical stage of cancer progression, remaining to be a major challenge in treatment of cancer and a leading cause of cancer-related mortality. The cascade of metastasis consists of a series of sequential steps, and cancer cells must complete before they can disseminate to secondary organs. After beginning with local invasion of adjacent tissue, the cancer cells invade the circulation through blood vessels or the lymphatic system, then disseminate to the different distant organs, and finally, they successfully colonize in the distant secondary organs.^13^

Evidence indicates that cancer-derived exosomes are participating in critical steps of the metastasis in the primary tumor. Exosomes promote tumor metastasis by enhancing cancer cells invasion and migration, reprogramming the extracellular matrix.^107^ Additionally, cancer-derived exosomes induce multiple cell populations and recruit them to secondary organ sites. The interactions among these exosomes, local stromal cells and cancer-recruited cells may create a favorable microenvironment for tumor metastasis.^7^ The establishment of pre-metastatic niche is the precondition that supports the distal settlement of cancer cells and promotes metastasis. Meanwhile, angiogenesis and lymphangiogenesis, immunosuppression, and reprogramming are all vital features of pre-metastatic niche.^108^

Cancer cells experience strong selective pressure during metastatic cascade, while metastatic cancer cells need to undergo heightened oxidative stress.^109^ Cells detachment induce a large amount of ROS, and excessive ROS can cause cell death, namely anoikis, which is an obstacle to metastasis.^110^ ROS is mainly derived from the oxidative metabolism of mitochondria. The Warburg effect may reduce the production of mitochondrial ROS, and increase glycolysis while also enhancing the pentose phosphate pathway to produce NADPH, which is vital for antioxidant activity.^111^ By limiting the oxidative metabolism of mitochondria, the “Warburg effect” decreases oxidative stress. Additionally, glucose is metabolized to lactate and pyruvate via glycolysis, and excess lactate production leads to acidic TME.^112^ In vivo study has shown that extracellular acidification favored metastasis of melanoma cells.^113^ Similarly, another study suggested that local stromal acidification promoted the formation of pre-metastatic niche in melanoma.^114^

It has been demonstrated that KSHV-infected cells transferred virus-encoded miRNAs to surrounding cells by exosomes, leading to a metabolic shift toward the glycolysis in the non-infected surrounding cells. In turn, the exosomes-mediated metabolic reprogramming of the non-infected cells promoted the growth of infected cells, thereby accelerating the migration and progression of Kaposi’s sarcoma.^106^ Human melanoma-associated exosomal miR-210 and miR-155 produced by melanoma were incorporated by normal stromal fibroblast cells, which led to increased aerobic glycolysis and decreased OXPHOS in fibroblasts, and finally resulting in extracellular acidification. Meanwhile, inhibition of miRNA activity reversed exosome-mediated metabolic reprogramming of stromal cells. Together, the stromal acidification contributed to creating the pre-metastatic niche via exosomes, and facilitated the tumor migration and invasion.^114^ Similarly, the LMP1 in EVs could activate the fibroblasts to CAFs through NF-κB/p65 pathway, and aerobic glycolysis was increased in activated CAFs. Furthermore, EVs packaged LMP1 markedly increased the important pre-metastatic niche factors (S100A8, VEGFR1 and fibronectin) levels by stimulating CAFs in vivo favored the establishment of pre-metastatic niche in nasopharyngeal carcinoma.^54^ Primary prostate cancer (PCa) cells transferred exosomes containing PKM2 into bone marrow stromal cell. Furthermore, CXCL12, an important promoter of bone metastasis in PCa, could be induced by an exosome-mediated HIF1α-dependent pathway, which created a pre-metastatic niche and promoted the progression of PCa metastasis.^80^ In breast cancer, large exosomes secreted from cancer cells were found to contain miR-122, which could remodel metabolism in the pre-metastatic niche to exacerbate metastasis.^115^ Breast cancer cells could suppress uptake of glucose by stromal cell in pre-metastatic niche of brain and lung, by transferring exosomes that carried enriched miR-122. Then, miR-122, incorporated by non-tumor cell in pre-metastatic niche, targeted PKM2 and inhibited glycolysis, which could reduce the utilization of glucose through niche cells and allow the cancer cell to utilize glucose. In addition, a study has found that exosomes containing VEGF could enhance glycolysis of ECs.^71^ VEGF signaling could loosen tight junctions between ECs, which induced vascular permeability and increased cancer cells transendothelial migration.^69^ Another study has also demonstrated that cancer-derived exosomes could remodel the ECs by destroying the function of endothelial cell barriers and inducing vascular permeability, which releasing cancer cells into the bloodstream for metastasis.^71^ These findings reveal that the non-tumor cells that take up the exosomes secreted by the cancer cells regulate the remodeling of metabolism, which promote the tumor metastatic capacity. Taken together, exosomes-mediated metabolic reprogramming may be a critical mechanism to exacerbate migration and metastasis.

### Tumor drug resistance

The development of tumor drug resistance remains a major barrier to the therapeutic effect. It is currently known that tumor drug resistance mechanisms include drug efflux, altered drug metabolism, altered energy programming, DNA damage repair, cancer stem cells, and epigenetic alterations.^116^

Evidence suggests that exosomes have emerged as critical player in the onset and development of tumor drug resistance.^117,118^ Because the drug susceptibility is different in tumor cells, exosomes secreted by drug-resistant tumor cells were transferred to drug-sensitive cells, which might also increase the resistance of the susceptible tumor cells.^119,120^

The metabolic reprogramming and drug resistance are closely correlative. The enhanced glycolysis of the tumor results in increased lactate and H^+^ production, which could lead to the acidosis of the TME. Moreover, the TME acidosis has been reported to contribute to drug resistance.^121,122^

Wang et al. demonstrated that exosomes secreted by chemoresistant colorectal cancer cells could be incorporated by chemosensitive cells, thereby enhancing the glycolysis and chemoresistance.^119^ Similarly, another study showed that exosomes with high acid SMase (ASM) content were able to transfer the phenotype of drug-resistant to drug-sensitive multiple myeloma cells through regulating lipid metabolism. After anti-myeloma drug stimulation, ASM expression and protein levels were increased in multiple myeloma cells and their exosomes, which reflected the tumor-protective effect of ASM and promoted the development of drug resistance.^120^ Moreover, Huang and colleagues^72^ indicated that AML-secreted exosomes increased glycolysis in ECs, and resulted in the remodeling of vascular and the acquisition of chemoresistance. Recently, Petanidis S et al.^122^ isolated exosomes from patients with Kras chemoresistant lung cancer patients.^122^ Exosomes can remodel metabolism in a PKM2-dependent manner to maintain lung cancer cell metabolic chemoresistance.^122^ Therefore, these data show that exosomes secreted by the cancer cells promote the drug resistance by remodeling the metabolism. In turn, targeting exosomes may become a new target for tumor drug-resistant therapy.

### Tumor immunosuppression

Cancer progression is intimately related to the dysregulation of immune cell subsets and chronic inflammation. In the environment of chronic inflammation, multiple complex mechanisms mediated by inflammatory cells contribute to cancer development. Inflammation is closely linked to immunity, and the same immune cells population causes both immune response and inflammation.^123^

Cancer cells-derived exosomes promote the induction of immunosuppression and inflammation, facilitating cancer progression. NKG2D, an activated cytotoxicity receptor, plays a vital role in immunosuppression in the tumor. Lundholm et al. identified that exosomes derived from prostate cancer cell expressed NKG2D ligands on their surface, which could induce the downregulation of NKG2D on natural killer (NK) cells and CD8 + T cells. The downregulation of NKG2D on natural killer (NK) cells and CD8 + T cells resulted in the impairment of their cytotoxic function, thereby promoting immunosuppression and tumor immune evasion.^124^ Furthermore, exosomes transfer immunosuppressive molecules from cancer cells to immune cells, which promote cancer progression by suppressing the function of immune cells.^125^ TGF-β is one of the major immunosuppressive cytokines. Exosomes derived from BC cells suppressed T cells proliferation through TGF-β, which inhibited immune response.^126^ A recent study demonstrated that human melanoma-secreted exosomes carried immunosuppressive programmed cell death ligand 1 (PD-L1), inhibited the function of CD8 T cells, and exacerbated tumor growth.^127^ Meanwhile, exosomes released by cancer cells also promote the mediation and persistence of inflammation. Exosomes-mediated pro-tumor inflammation through Toll-like receptor (TLR) signaling. For example, lung cancer cells secreted exosomal miR-21 and miR-29a, which could bind to TLR8 and stimulate the activation of NF-κB, leading to product the pro-inflammatory cytokines and promote tumor proliferation and metastasis.^128^ Exosomes containing hY4 derived from chronic lymphocytic leukemia (CLL) could be transferred to monocytes leading to key CLL-related phenotypes, including the release of the cytokines IL-6, CCL2, CCL4, and the promotion of PD-L1 expression. Moreover, exosomes-mediated transferred hY4 to monocyte facilitate cancer-related inflammation and immune escape through the upregulation of PD-L1.^129^

It has been shown that cancer cells with high glycolytic activity have a strong ability to escape immune surveillance, due to T-cells are impaired in their ability to kill cancer cells. However, inhibition of glycolysis improved the anti-tumor immunity of T cells.^130^ Meanwhile, the glycolytic metabolite, lactate, could directly inhibit the cytolytic activity of NK cells and indirectly inhibit NK cells function by increasing the number of myeloid-derived suppressor cells (MDSCs).^131^ In addition to glucose metabolism, amino acid metabolism is also recognized to play a vital role in immunosuppression. Arginine (Arg) metabolism is an important pathway in regulating immune cell reactivity, and two key enzymes are involved in the mechanism of immune escape: nitric oxide synthase (NOS) and arginase (ARG). Overexpression of NOS or ARG in tumor cells leads to Arg consumption in the TME and inhibits T-cells proliferation and function.^132^ Glutamine exhaustion by clear cell renal cell carcinoma (ccRCC) cells induce macrophages to secrete IL-23 via the activation the HIF1α pathway. IL-23 activates regulatory T cells (Treg) and promotes the expression of IL-10 and TGF β, thereby inhibiting T-cell cytotoxic activity and mediate immune escape.^133^ In lipids metabolism, fatty acid synthase (FASN) is a critical metabolic enzyme in the de novo synthesis of fatty acids. Jiang et al.^134^ found that the overexpression of FASN in ovarian cancer leads to lipids accumulation in the TME, causing T cells dysfunction, which in turn induces impaired anti-tumor immune response. In the TME, cancer cells product metabolic byproducts by metabolic reprogramming, including lactate, ROS, nitric oxide, prostaglandin, and arachidonic acid, resulting in an inflammatory microenvironment.^135^ Inflammatory cells may influence, and be influenced by, cancer cells metabolism, primarily glucose metabolism, and promote tumor growth and immune escape through metabolic pathways.^123^

A recent study by Basso et al. reported the effects of exosome-mediated metabolic reprogramming on tumor immunosuppression. Pancreatic ductal adenocarcinoma (PDAC)-secreted exosomes from cells without the expression of SMAD4 could create an immunosuppressive myeloid cells background by increasing glycolysis and calcium flux through transferring differentially expressed miRNA and protein associated with SMAD4.^136^ These results suggested that immunosuppression induced by exosome-mediated metabolic reprogramming could be a potential inducer of tumor progression.

## The underlying molecular mechanism of exosome-mediated metabolic reprogramming in TME remodeling

In the intercellular communication, exosomes mediate the exchange of biologically active molecules between cells and remodel the biological characteristics of recipient cells. Recently, accumulating evidence has shown that exosomes have been recognized as crucial mediators in the metabolic reprogramming of cancer cells and stromal cells. To determine how exosomal cargoes influence the metabolic reprogramming in the TME, in this section, we will discuss the contents in exosomes from cancer cells and stromal cells in the TME (Table 1), such as exosomal proteins, miRNAs, ncRNAs, and metabolites, as well as the underlying mechanisms by which exosomes affect metabolic reprogramming.

**Table 1:** Overview of exosomal cargoes and functions in cancer

Cargoes type	Exosomal cargoes	Donor cells	Recipient cells	Function	Ref.
Protein	PKM2, GLUT1	LPS-activated LX-2 and activated primary HSCs	HSCs, KCs, and LSECs	Induces the glycolysis of quiescent HSCs LSECs and KCs	^138^
	PKM2	LNCaP, PC3, and C4-2B	ST2, mouse BMSCs	Creates a pre- metastatic niche through transferring PKM2	^80^
	VEGF	HL-60, U937, primary AML cells	HUVECs	Promotes chemoresistance by inducing the glycolysis in HUVECs	^72^
	ALDOA, ALDH3A1	Irradiated A549 and NCI-H446	Unirradiated A549 and NCI-H446	Enhances the motility of the recipient cells by promoting the glycolysis	^97^
	LMP1	CM	CAFs	Increases the glycolysis and autophagy in CAFs	^54^
	ITGB4	MDA-MB-231, BT-20	CAFs	Promotes the glycolysis and the export of lactate in CAFs	^52^
	FAO-related proteins	Adipocytes	SKMEL28 and 1205Lu	Promotes tumor aggressiveness by inducing fatty acid oxidation	^67^
	AM	PC patient-derived cell lines	Adipocytes	Induce lipolysis	^139^
miRNA	miR-105	MCF10A and MDA-MB-231	NIH3T3 and WI-38	Promotes tumor growth by reprogramming the metabolism of CAFs	^53^
	miR-122	MCF10A and MDA-MB-231	Mouse lung fibroblasts and Mouse astrocytes	Remodels metabolism of the niche cells to promote tumor progression	^115^
	miR-155	4 T-1, C2C12 and HEK 293T	3 T3-L1	Induces the beige/brown differentiation and promotes lipolysis in the adipocytes	^64^
	miRNAs	KSHV-infected LECs	HUVEC and LEC	Promotes the glycolysis in the non-infected cells.	^106^
	miR-155, miR-210	1770-Her4, 2183-Her4, 1300-mel, HMCB, 526-mel, 888-mel, and Hs 294T	HADF	Promotes the tumor metastasis through enhancing the glycolysis in stromal cells	^114^
	miR-126, miR-144	MCF-7, MDA-MB-231, and HEK 293T	3T3-L1	Inducing the metabolic reprogramming in adipocytes to promote tumor progression	^65^
lncRNA	SNHG3	Breast cancer patient-derived fibroblast cells	MCF-7 and MD-MBA-453	Promotes the tumor growth by reprogramming the metabolism of breast cancer cells	^98^
	HISLA	TAMs	MDA-MB-231, MDA-MB-468, BT-474, and MCF-7	Enhances the glycolysis and chemoresistance of breast cancer cells	^93^
circRNA	ciRS-122	SW480/oxaliplatin (L-OHP) and HCT116 /L-OHP	SW480 and HCT116	Increases the glycolysis and decreases drug sensitivity in the sensitive cancer cells	^119^
Metabolite	Lactate, glutamate	hMSCs	MCF-7	Promote the tumor growth	^144^

### Exosomal proteins

Proteins are the main components in exosomes. Of note, previous proteomic study showed that glucose metabolism-associated proteins were enriched in exosomes from cancer cells.^137^ The GLUT1 affects the absorption and utilization of glucose, which is a rate-limiting transporter for glucose uptake. PKM2 is a glycolytic enzyme that has been recognized as crucial mediator in the glycolytic pathway. In addition, several studies have found that exosomes contained GLUT1 and PKM2.^80,138^ Wan et al.^138^ showed that enriched PKM2 and GLUT1 were secreted in exosomes from activated hepatic stellate cells (HSCs), which induced the “Warburg effect” of quiescent HSCs, Kupffer cells (KCs) and liver sinusoidal endothelial cells (LSECs); meanwhile, inhibition of Hif-1 by 2-ME (an inhibitor of Hif-1) or specific siRNA suppressed the expression of PKM2 and GLUT1 in exosomes from LPS- or hypoxia-activated LX-2 cells. Another study showed that prostate cancer-secreted exosomes transferred PKM2 protein to stromal cells rather than inducing PKM2 mRNA synthesis or protein synthesis, which increased CXCL12 production in the stromal cells to create a pre-metastatic niche.^80^ VEGF is an important angiogenesis factor. The study from Wang et al.^72^ showed that VEGF transferred by AML cells derived exosomes activated VEGF signaling pathway in HUVECs, resulting in inducing proliferation and tube formation in HUVECs through promoting glycolysis, in turn, which promoted the chemoresistance of AML cells. Shao and colleague^97^ demonstrated that metabolic enzymes ALDOA and ALDH3A1 were increased in exosomes released by irradiated lung cancer cells, and exosomes secreted from irradiated cancer cells transferred the metabolic enzymes to the unirradiated cancer cells, which enhanced the motility of the recipient cells through accelerating glycolysis. Moreover, EVs packaged LMP1 secreted by nasopharyngeal carcinoma (NPC) activated normal fibroblasts into CAFs through the NF-κB/p65 pathway, and increased aerobic glycolysis and autophagy in activated CAFs. Meanwhile, the CAFs upregulated the expression of MCT4 through activation of p65 pathway, which could export β-HB and lactate to MCT1-expressing cancer cells. The uptake of β-HB and lactate by cancer cells enhances the OXPHOS, which could promote the tumor progression and induce radiation resistance of tumor both in vitro and in vivo.^54^ Integrin beta 4 (ITGB4) overexpression has emerged as critical player in cancer progression. Sung and colleagues^52^ found that the triple-negative breast cancer (TNBC) cells-derived exosomal ITGB4 proteins induced BNIP3L-dependent mitophagy and enhanced expression of MCT4, which promoted the glycolysis and the export of lactate in CAFs. Some studies have found FAO-related proteins in adipocytes-derived exosomes through proteomics analysis, and these exosomes could induce metabolic reprogramming in melanoma cells for the benefit of FAO.^67,68^ Another study showed pancreatic cancer (PC) cells-secreted exosomes containing adrenomedullin (AM) interacted with adrenomedullin receptors (ADMRs) in adipocytes, and activated MAPKs p38 and ERK 1/2 signaling pathways and induced lipolysis through HSL phosphorylation.^139^

### Exosomal microRNAs

Recently, exosomal microRNAs have been implicated in intercellular communication. Yan et al. observed that exosomal miR-105 derived from breast cancer cells could activate MYC pathway in CAFs to induce a metabolic reprogramming. Moreover, CAFs could display different metabolic profiles depending on the metabolic environment. When nutritionally adequate, the exosomal miR-105 reprogrammed CAFs with enhanced glycolysis and glutaminolysis, thereby providing fuel to the neighboring cancer cells. When at a low nutrient level and metabolic byproduct accumulate, CAFs converted metabolic waste products (including lactate and ammonium) into energy-rich metabolites, which contributed to promoting tumor growth.^53^ Fong and collaborators found that breast cancer cell-secreted exosomal miR-122 could suppress the glucose uptake by the niche cells through downregulating PKM2 and GLUT1. Meanwhile, inhibition of exosomal miR-122 could rescue glucose metabolism in distant organs, such as lungs and brain, and decreased the incidence of tumor metastasis in vivo, which demonstrated that exosomal miR-122 was able to remodel systemic energy metabolism to promote tumor progression.^115^ In another study, Wu et al. showed that miR-155 directly targets peroxisome proliferators-activated receptor γ (PPARγ). The exosomal miR-155 derived from breast cells mediated the beige/brown differentiation and promoted lipolysis in the adipocytes by downregulating the expression of PPARγ. In contrast, glycolysis was not significantly influenced by the exosomal miR-155 in the cancer cells. This study also proved that cancer cells co-cultivated with mature adipocytes stimulated the aggressive phenotype by inducing EMT. It could be speculated that exosomal miR-155 was associated with the tumor-promoting process.^64^ A study from Yogev et al.^106^ reported that KSHV-infected cells transferred the miRNAs to surrounding cells through secreting exosomes, which resulted in a metabolic reprogramming toward the glycolysis, decreased mitochondrial biogenesis and the stabilization of HIF1α in the non-infected cells. In addition, Shu et al.^114^ observed that exosomes secreted by human melanoma cells, including enriched miR-155 and miR-210, remodeled stromal cells metabolism and might induce formation of pre-metastatic niche to promote tumor metastasis by enhancing aerobic glycolysis and suppressing OXPHOS in stromal cells. Wu and colleagues found that IRS1 is the direct target of miR-126, and the exosomal miR-126 derived from breast cancer cells induced metabolic reprogramming in adipocytes, including inducing the catabolism after the disruption of IRS/Glut-4 signaling pathways, activating the AMPK/autophagy pathways and stabilizing the expression of HIF1α. Meanwhile, this study also demonstrated breast cancer cells-secreted exosomal miR-144 mediated the beige/brown differentiation of adipocyte via the MAP3K8/ERK1/2/PPARγ axis. Moreover, inhibition of miR-126 or miR-144 decreased adipocyte-induced tumor progression in vivo, which revealed exosomal miR-126 and miR-144 from the tumor-adipocyte crosstalk remodeled systemic energy metabolism to promote tumor progression.^65^

### Exosomal lncRNA and circRNA

In addition to microRNAs, other non-coding RNA (ncRNA) classes such as lncRNAs and circRNAs have been investigated. Li et al. found that CAFs-derived exosomal lncRNA SNHG3 remodeled the metabolism after breast cancer cells uptake exosomes. In addition, SNHG3 acted as a sponge of miR-330-5p. Moreover, miR-330-5p targeted PKM and regulated by SNHG3 in breast cancer cells. Mechanistically, knockdown of exosomal SNHG3 suppressed breast cancer growth and glycolysis in vivo and in vitro through up-regulating miR-330 and decreasing the expression of PKM. These results demonstrated that CAFs-released exosomal lncRNA could remodel breast cancer cell metabolism and promoted the tumor growth.^98^ Another study from Chen and collaborators reported TAMs enhanced the apoptosis resistance and the glycolysis in breast cancer cell through transmitting EVs-packaged lncRNA HISLA. It had also been shown that blocking TAMs-secreted HISLA in EVs, which inhibited chemoresistance and aerobic glycolysis of breast cancer in vivo. In addition, lncRNA HISLA bound prolyl hydroxylase domain 2 and inhibited its interaction with HIF-1α, which prevented hydroxylation and degradation of HIF-1α. Intriguingly, lactates exported from cancer cell upregulated HISLA in TAMs and promotes the feed-loop between TAMs and cancer cells.^93^ Recently, Wang et al. showed that oxaliplatin-resistant colorectal cancer cells-derived exosomal ciRS-122 was a sponge of miR-122, which enhanced PKM2 protein expression, thus increased the glycolysis and decreased the drug sensitivity in the sensitive cancer cell. Meanwhile, it also indicated that exosomal si-ciRS-122 reversed the oxaliplatin resistance through inhibiting the ciRS-122/miR-122/PKM2 axis in vivo and in vitro.^119^

### Exosomal metabolites

Exosomal metabolites have also emerged as important players in tumor progression. Although the exosomal contents of are similar to the donor cells, they are enriched in certain lipids, including cholesterol, phosphatidylinositol, and phosphatidylcholine, and act as lipid mediators of intercellular transmission.^140^ Moreover, the enrichment of specific lipids in exosomes significantly increases the membrane stiffness of the exosomes. These lipids are present in the outer membrane of the exosome, and play a crucial role in the recognition and internalization of the exosome, which allows the exosomes to deliver the metabolites to the recipient cells.^141^ The exosomes carry bioactive lipids, such as leukotrienes and prostaglandins, which promote cancer progression.^142^ Moreover, exosomes derived from cancer cells carry glycosidases, which can cleave the extracellular matrix (ECM) components, such as glycoproteins and proteoglycans, resulting in ECM remodeling and facilitating tumor development.^143^ A metabolomics study showed glutamate and lactate in the EVs of human mesenchymal stem cells (hMSCs). Glutamate could provide precursors for the major macromolecular classes via carbon and nitrogen trafficking, and lactate could enhance the ability of cancer cells to survive in the hypoxic and nutrient-deficient conditions.^144^

## The potential application of exosome

### Exosome targeting metabolic reprogramming as diagnostic biomarker

Increasing evidence shows that exosomes hold enormous potential for diagnosis, prognosis, and evaluation of efficacy in tumor. Exosomes are easily obtained from most body fluids, including blood, saliva, urine, and ascites, which can act as promising biomarkers in tumor “liquid biopsy”.^9^ The exosomal cargoes are encapsulated by a lipid bilayer membrane, which protects the exosomal contents to prevent them from degrading during transportation. The biologically active molecules contained in exosomes reflect the different pathological states of cells origin and can remodel the metabolism of the recipient cells, therefore providing a rich source of biomarkers (Table 2).

**Table 2:** Metabolism-related exosome from biofluids as diagnostic biomarkers in cancer

Biomarker	Cancer type	Biofluids	Method	Clinical value	Ref.
ciRS-122 and miR-122	Colorectal cancer	Serum	Western blot, RT-qPCR	Expression levels of ciRS-122 are positively correlated with drug resistance.	^119^
HISLA	Breast cancer	Plasma	qRT–PCR	Diagnostic biomarker of chemoresistance for breast cancer.	^93^
AM	Pancreatic cancer	Plasma	Western blot	Diagnostic biomarker for early detection of PC	^139^

Wang et al. discovered exosomal ciRS-122 from the serum of oxaliplatin-resistant colorectal cancer patients and oxaliplatin-sensitive patients. Compared with the oxaliplatin-sensitive group, the oxaliplatin-resistant group had higher ciRS-122 expression levels in the exosomes. Moreover, Exosomal ciRS-122 derived from drug-resistant colorectal cancer cells could enhance the glycolysis to reduce drug sensitivity in drug-sensitive cells, which revealed that exosomal ciRS-122 could regulate metabolism to favor tumor drug resistance.^119^ In our opinion, exosomal ciRS-122 targeting metabolism might be a candidate for predicate tumor drug resistance in colorectal cancer. Recently, Chen et al. found that the expression level of EVs-packed lncRNA HISLA in plasma of breast cancer patients is closely correlated with the level of HISLA in tumor-associated macrophages isolated from the breast cancer samples. In addition, HISLA expression level was associated with glycolysis, poor chemotherapy effect, and low survival rate of breast cancer patients, indicating EVs-packed HISLA as an independent prognostic biomarker in breast cancer patients.^93^ Another study revealed that adrenomedullin (AM) was significantly upregulated in plasma exosomes from patients with pancreatic cancer (PC) compared to non-PC control patients. Moreover, PC-exosomal AM induced lipolysis in subcutaneous adipocytes of PC patients demonstrated that exosomal AM could be a potential early diagnostic biomarker of pancreatic cancer patients.^139^ These findings suggest that the exosomes may be concerned as promising diagnostic and prognostic biomarkers for cancer therapy by targeting metabolic reprogramming.

### Exosome-mediated metabolic reprogramming as therapeutic target

The specific biological contents of exosomes profoundly facilitate the cancer progression. Thus, the current therapeutic strategies mainly focus on inhibiting the production and secretion of exosomes, blocking exosome-mediated intercellular communication, and eliminating the specific exosomal active molecular. The nSMase2 inhibitor, GW4869, is widely used to suppress the secretion of exosomes, which significantly inhibit the production of exosomes.^145^ There is growing evidence that metabolic reprogramming is an important mechanism influencing cancer progression. Therefore, finding an effective way to inhibit metabolic reprogramming and thus treat cancer is a potential new approach.

As discussed in this paper, the latest evidence confirms that the exosomal pathway is important for tumor metabolic reprogramming. So, we speculate that targeting regulation of exosomal-related mechanisms may be able to treat cancer by affecting metabolic reprogramming. Recently, Li et al. demonstrated that CAFs-derived exosomes enhanced glycolysis, inhibited mitochondrial OXPHOS, and promoted the proliferation of breast cancer cells. However, the GW4869 could reverse the metabolic changes of breast cancer cells, which in turn led to inhibit the cancer progression.^98^ Another study also showed that the secretion of exosomes was blocking by GW4869, which inhibited the glycolysis and activation of recipient cells.^138^ Wang et al.^119^ found that exosomes could act as gene delivery vesicles transferring short interfering RNA (siRNA) to target cancer cells, which inhibited glycolysis and reversed oxaliplatin resistance in drug-resistant colorectal cancer cells. According to this biological characteristic of exosomes, Shu et al.^114^ discovered that exosomes transferred with miRNA inhibitors could reverse the exosome-mediated metabolic reprogramming, which could reduce the risk of tumor metastasis. Notably, exosomes have multiple advantages as potential therapeutic targets owing to their crucial role during tumor progression. Although many in vitro studies targeting exosomes have been performed to treat cancers, few clinical trials have been conducted. We speculate that targeted therapy aiming to suppress the production and transfers of exosomes would be a potent way to inhibit tumor progression, especially tumor metastasis. Targeting exosomes combined with targeting cancer cells have a promising effect on inhibiting cancer progression, which may be a potential treatment strategy for future tumor treatment.

## Conclusion

Exosomes secreted from cancer cells and their surrounding stromal cells can be incorporated by the recipient cells to communicate with each other, which results in metabolic changes in the TME. As mentioned above, exosomes are implicated in metabolic reprogramming and microenvironment remodeling, which profoundly exacerbate tumor angiogenesis, metastasis, and drug resistance. The existing researches have partially clarified the mechanism of exosomes in the TME, mainly attributed to the specific biologically active substances they carry. In particular, in the TME, there seems to be a bi-directional transfer of cargos between cancer cells and stromal cells. Exosomes derived from cancer cells remodel the metabolism of stromal cells, thereby facilitating cancer progression; on the other hand, cancer-associated stromal cells secreted exosomes that can also affect tumor development by regulating cancer cells metabolism. Furthermore, current clinical studies have focused on the development of exosomes, and through its relationship with metabolism in the TME, exosomes are recognized as early diagnostic and prognostic biomarkers, and potential targets of the cancer therapy. Despite advancements in exosome research, there are still some problems that need to be addressed. First of all, the isolation and purification of exosomes are still challenging, and the separation technology needs to be continuously proposed and improved to increase its yield and purity. Second, it is unclear whether cancer cells consistently secrete similar amounts and levels of exosomes or whether they change with cancer progression, and how cancer cells regulate exosomes secretion rate. How do exosomes accurately transfer these active substances to specific receptor cells and the underlying mechanisms that affect metabolism still needs further elucidation. Additionally, there are few studies on exosomes-mediated lipids and amino acids metabolism. Notably, further studies focus on lipids and amino acids metabolism to understand how exosomes carry metabolites to facilitate tumor progression. Third, exosome is currently considered a promising biomarker for diagnosis, prognosis and therapeutic effect of cancer therapy. However, current clinical studies usually have small samples and poor reproducibility, so large multi-center studies are needed to improve the liquid biopsy effectiveness. Finally, the biological characteristics of exosomes and the active substances they deliver make them attractive targets for cancer therapy. Future research should focus on identifying cargoes whose functions can be used to design effective therapies. Moreover, whether therapies targeting exosomes could be effective in preventing tumor metastasis? However, the application of exosomes in anti-tumor therapy still has a long way to go.
